# Supplementary material for: Type One Protein Phosphatase 4aD Negatively Regulates Cotton (Gossypium hirsutum) Salt Tolerance by Inhibiting the Phosphorylation of Kinases That Respond to Abscisic Acid
Source: Int J Mol Sci. 2025 Apr 8;26(8):3471. doi: 10.3390/ijms26083471 (PMC12026917; doi:10.3390/ijms26083471)
Supplement: Supplementary file 1 [file ijms-26-03471-s001.zip › 2025.04.05 Supplementary Figures.pdf]

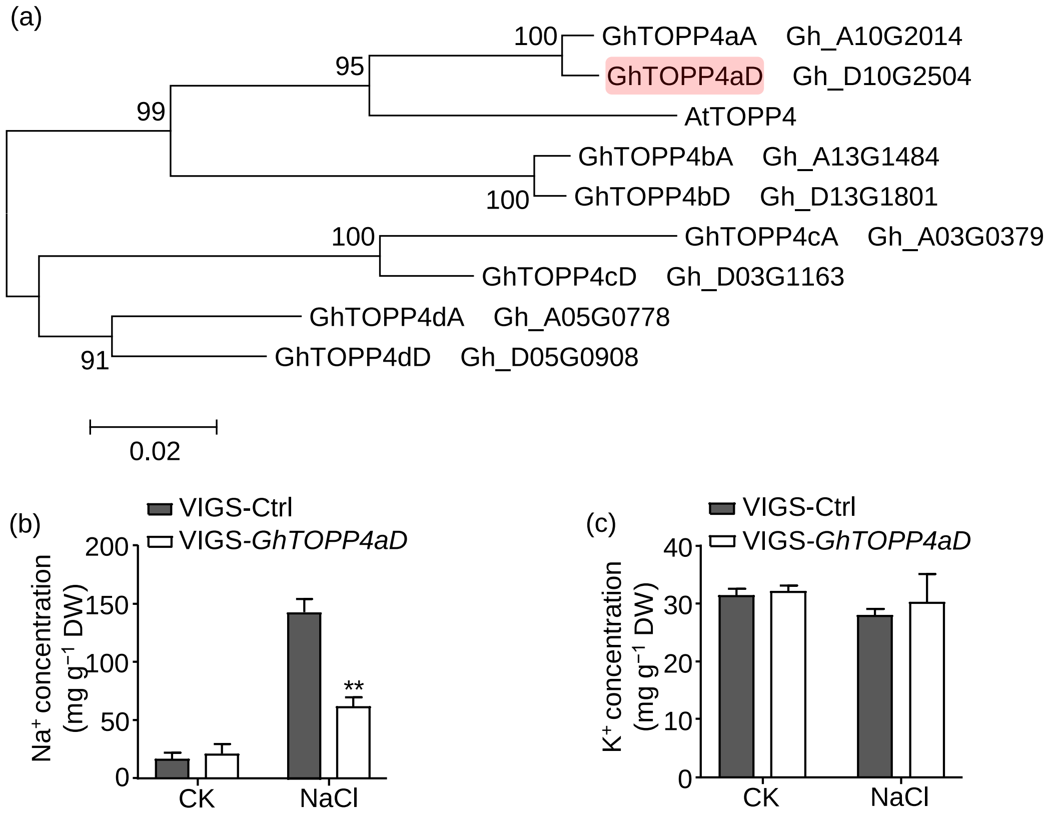

Fig.S1 GhTOPP4aD negatively regulates cotton salt response.

(a) Phylogenetic tree of *TOPP4* family in both cotton and Arabidopsis. The amino acid sequences of TOPP4 in both cotton and Arabidopsis were collected to generate the phylogenetic tree using the neighbor-joining method in MEGA v.12. The pink color indicates the name of the gene used in this article. (b, c) Na^+ and K^+ content in VIGS-Ctrl and VIGS-*GhTOPP4aD*. With 300 mM NaCl treatment for 3 days after VIGS was established, 0.3 g cotton leaves were digested with 1M HCl for 24 h, and the ion accumulation from cotton leaves was measured using an atomic absorption spectrophotometer 'see the Materials and Methods section'. The data are shown as means ±SD from three independent repeats(n = 3; **, p < 0.01, Student's t-test).

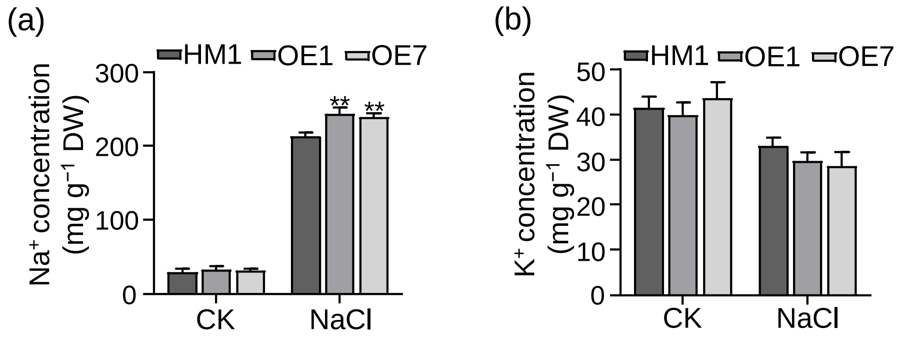

Fig.S2 Overexpression of *GhTOPP4aD* in Cotton increases the Na^+ contents in response to salt stress. (a, b) Na^+ and K^+ content in HM1 and OE-*GhTOPP4aD*. With 300 mM NaCl treatment for 3 days, 0.3 g cotton leaves were digested with 1M HCl for 24 h, and the ion accumulation from cotton leaves was measured using an atomic absorption spectrophotometer 'see the Materials and Methods section'. The data are shown as means ±SD from three independent repeats(n = 3; *, $p < 0.05$, **, $p < 0.01$, Student's t-test).

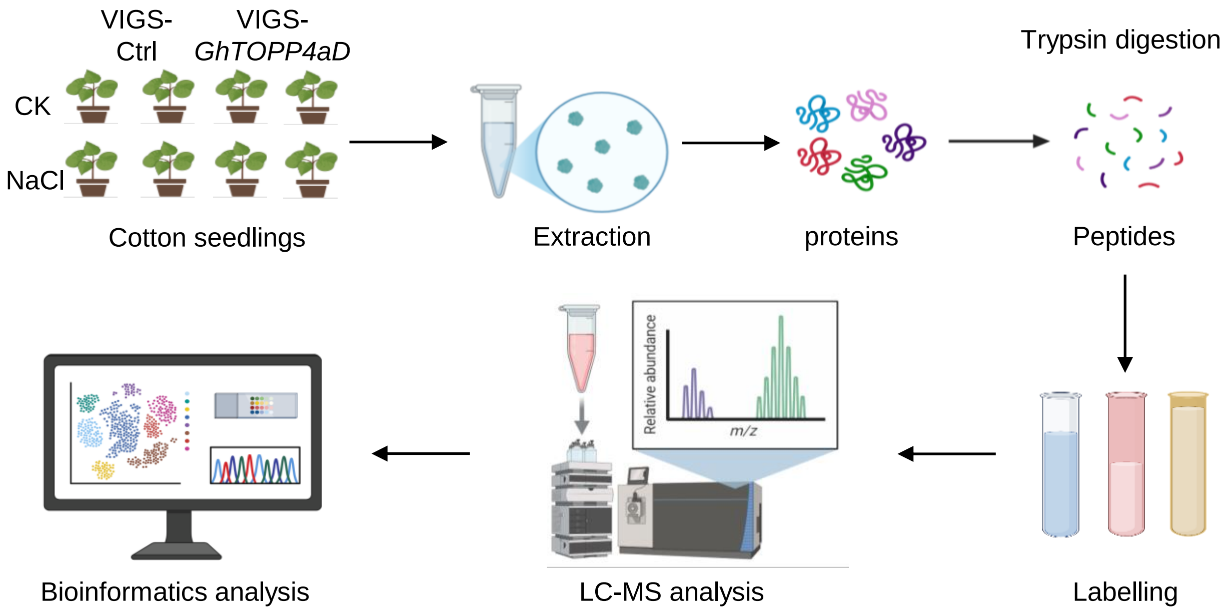

Figure S3 Workflow for phosphoproteome profiling of cotton seedlings under salt stress.

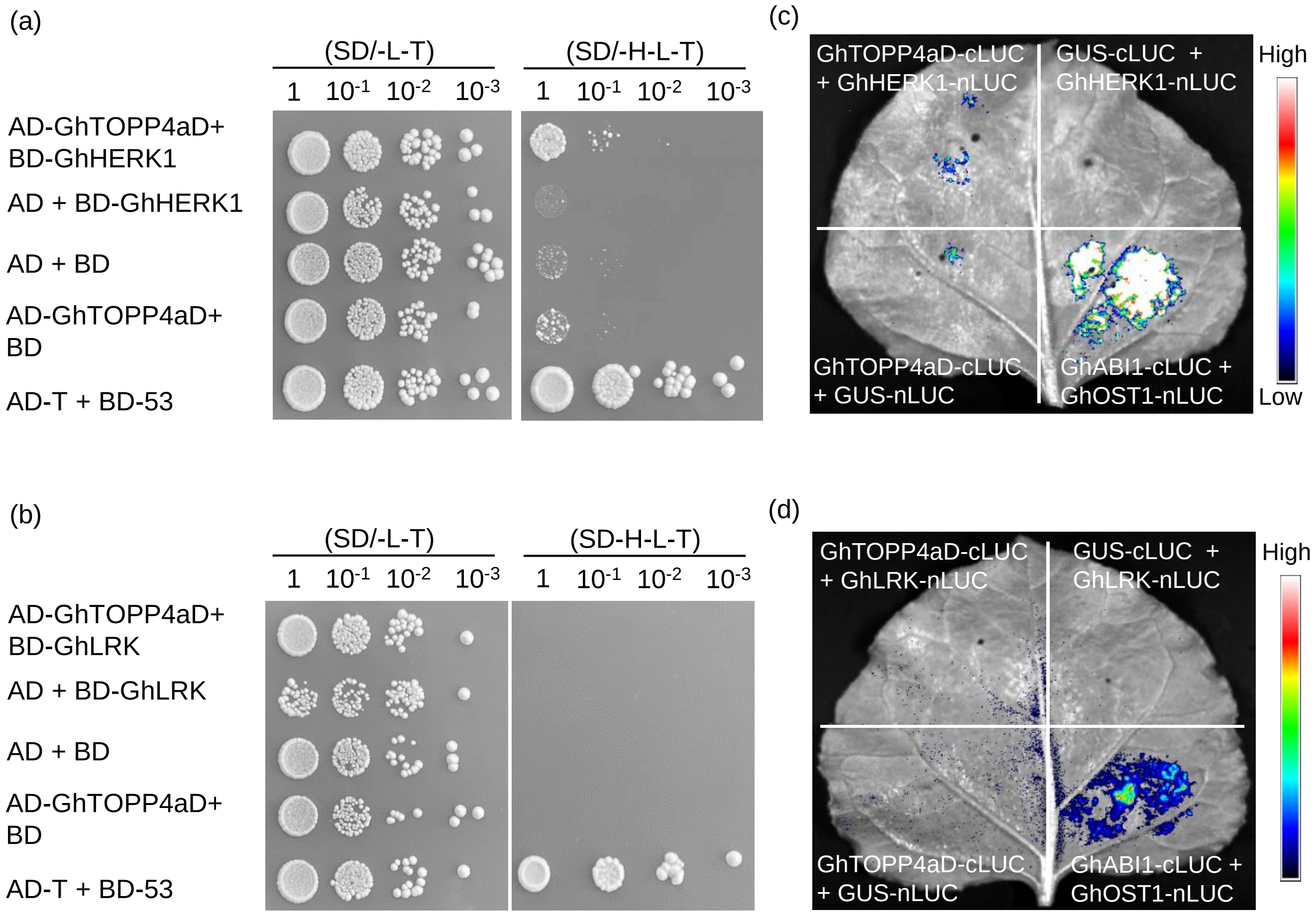

Fig.S4 Analyze the interaction between GhTOPP4aD and GhHERK1 or GhLRK using yeast two-hybrid (Y2H) and luciferase complementation imaging (LCI) assays. (a-b) Yeast two-hybrid (Y2H) assay showing the interaction between GhTOPP4aD and GhHERK1 or GhLRK. SD/-L-T, synthetic medium without Trp and Leu; SD/-H-L-T, synthetic medium without Trp, Leu, His. DNA binding domain (BD) and activation domain (AD) were used as empty controls. (c-d) LUC complementation imaging (LCI) assay was used to assess the binding between GhTOPP4aD and GhTOPP4aD and GhHERK1 or GhLRK. Representative images of *N. benthamiana* leaves 48 h after infiltration are shown. The bar showing red to blue indicates luciferase signal intensity from high to low.

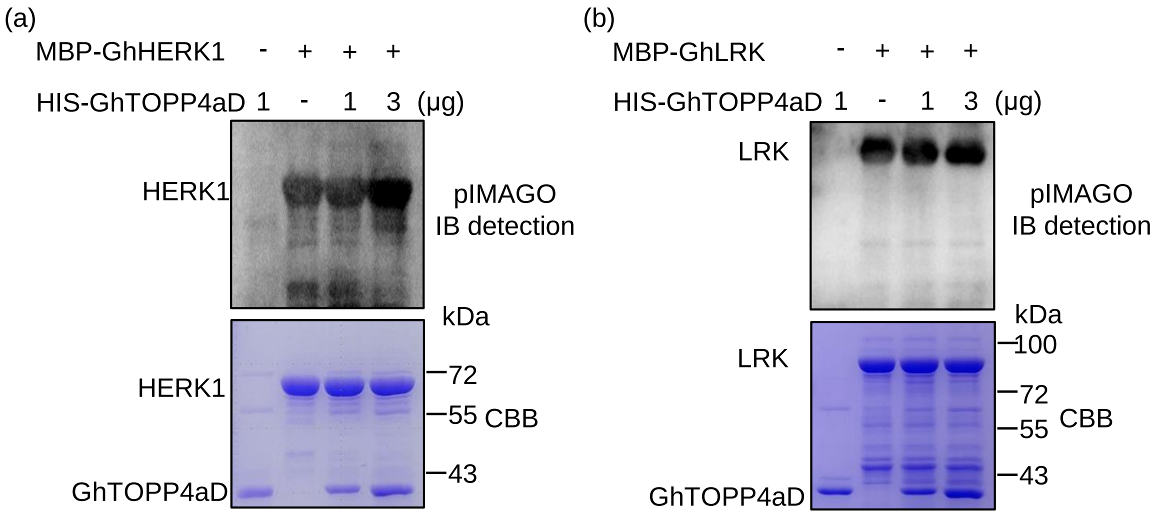

Fig. S5 Western-blot analysis the dephosphorylation regulatory relationship of GhTOPP4aD towards tyrosine kinases through dephosphorylation assays *in vitro*. (a-b) Analyze the phosphorylation status of tyrosine kinases through *in vitro* dephosphorylation assays. Recombinant proteins of MBP-GhHERK1, and MBP-GhLRK were incubated with a gradient concentration of His-GhTOPP4aD for the dephosphorylation assay at 30℃ for 30 min and separated by 12.5% SDS-PAGE, respectively.

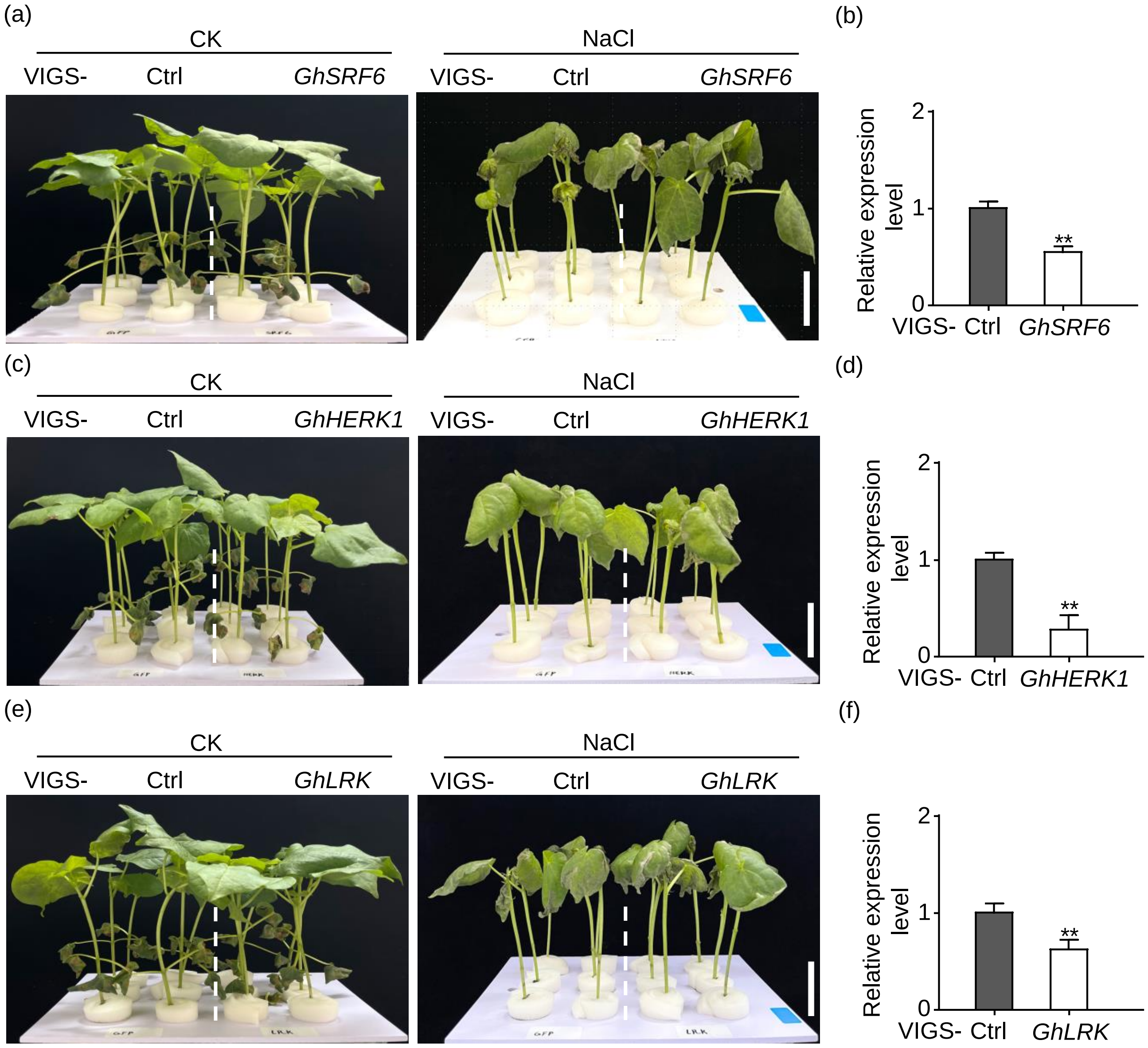

Fig. S6 Salt stress-related phenotypes in VIGS-*GhSRF6*, VIGS-*GhHERK1*, and VIGS-*GhLRK* plants. (a-f) The phenotypes of VIGS plants with or without NaCl treatment and their corresponding relative expression levels. Fourteen-day-old silenced plants were subjected to 300 mM NaCl for 3 d. The dashed lines separate different VIGS groups. Bar = 3 cm. The leaf samples from VIGS plants were collected to detect the expression of *GhSRF6* (b), *GhHERK1* (d), and *GhLRK* (f) without NaCl treatment by real-time quantitative PCR (RT-qPCR). GhActin9 was used as the internal control. The data are shown as means ±SD from three independent repeats (n = 3; **, $p < 0.01$, Student's t-test).
